# Rabies Virus Infection in *Eptesicus fuscus* Bats Born in Captivity (Naïve Bats)

**DOI:** 10.1371/journal.pone.0064808

**Published:** 2013-05-31

**Authors:** April D. Davis, Jodie A. Jarvis, Craig Pouliott, Robert J. Rudd

**Affiliations:** Rabies Laboratory, Wadsworth Center, New York State Department of Health, Slingerlands, New York, United States of America; Metabiota, United States of America

## Abstract

The study of rabies virus infection in bats can be challenging due to quarantine requirements, husbandry concerns, genetic differences among animals, and lack of medical history. To date, all rabies virus (RABV) studies in bats have been performed in wild caught animals. Determining the RABV exposure history of a wild caught bat based on the presence or absence of viral neutralizing antibodies (VNA) may be misleading. Previous studies have demonstrated that the presence of VNA following natural or experimental inoculation is often ephemeral. With this knowledge, it is difficult to determine if a seronegative, wild caught bat has been previously exposed to RABV. The influence of prior rabies exposure in healthy, wild caught bats is unknown. To investigate the pathogenesis of RABV infection in bats born in captivity (naïve bats), naïve bats were inoculated intramuscularly with one of two *Eptesicus fuscus* rabies virus variants, EfV1 or EfV2. To determine the host response to a heterologous RABV, a separate group of naïve bats were inoculated with a *Lasionycteris noctivagans* RABV *(LnV1)*. Six months following the first inoculation, all bats were challenged with EfV2. Our results indicate that naïve bats may have some level of innate resistance to intramuscular RABV inoculation. Additionally, naïve bats inoculated with the LnV demonstrated the lowest clinical infection rate of all groups. However, primary inoculation with EfV1 or LnV did not appear to be protective against a challenge with the more pathogenic EfV2.

## Introduction

Worldwide, more than 70,000 people die of rabies every year [1]. In undeveloped and developing nations, 95% of all human rabies deaths are the result of infection with a canine rabies variant. Conversely, human rabies cases in developed countries are typically the result of a chiropteran lyssavirus [2], [3]. The most common rabies virus variant (RABV) associated with human deaths in the United States is *Lasionycteris noctivagans* (silver-haired bat) RABV [4]. The silver-haired bat is considered to be a migratory, tree roosting species, rarely encountered by humans [5], [6]. In contrast, the bats most commonly encountered by humans in the United States are the colonial bats *Eptesicus fuscus*, *Tadarida brasiliensis*, and *Myotis spp*. [7], [8]. These bats are often found living in the same dwelling as humans in numbers ranging from a few individuals to well over a thousand bats. It is unclear why human rabies cases are typically the result of a bat RABV circulating in solitary bats as opposed to colonial bat species with frequent human contact [9], [10]. Previous studies have suggested that the *Lasionycteris noctivagans* variant is more pathogenic than other rabies virus variants [11], [12]. Morimoto et al. (1996) reported the ability of the *L. noctivagans* RABV (LnRV) to replicate at lower temperatures and in non-neuronal cell types when compared to a canine RABV. However, it is unknown if LnRV is more pathogenic in vitro and vivo when compared to other bat RABV, and the capacity of LnRV to spill over into a colonial heterologous host species has not been well studied.

The ability to study RABV in bats can be problematic as bats brought into research colonies are wild caught. Previous research has documented the presence of naturally acquired antibodies in wild caught bats ranging from 0 to 63%, depending on the bat species and location of the study [13]–[17]. Additionally, the presence of circulating anti-rabies antibodies in bats appears to be transitory [16], [17]. Following RABV inoculation of wild-caught bats, the presence of circulating anti-rabies neutralizing antibodies (VNA) was detected 13 days post inoculation and bats that survived the inoculation were seronegative by day 139 post-inoculation [16], [17]. With this knowledge, it is difficult to determine if a seronegative, wild caught bat has been previously exposed to rabies. In wild caught bats, it is unknown what effect prior RABV exposure may have on experimental results.

The study of rabies in bats is multifactorial and the outcome may be based on previous exposure, variant to which the animal is exposed, location of exposure, dose, age, and the bats genetic background. Unlike mouse studies, bats are wild caught and thus likely to differ both genetically, in health, and in age. The diversity among bats in a study may account for the unpredictability in response to RABV inoculation. Turmelle et al (2010) reported variability in the mortality and development of VNA in *E. fuscus* following i.m. inoculation with an *E. fuscus* RABV [17]. A separate study by Davis et al 2012, supports their finding: 100% of *E. fuscus* developed rabies following i.m. inoculation with 10^3^ TCID_50_ whereas 40% developed rabies following inoculation with the same RABV at 10^2^ TCID_50._

The purpose of this study was to determine if bats born in captivity (naïve bats) with no prior exposure to rabies would be highly susceptible to RABV infection following experimental inoculation. Furthermore, to ascertain the effect of a heterologous RABV, one group of naïve bats was inoculated with LnRV. Our results indicate naïve bats may be marginally more likely to develop clinical rabies virus infection following intramuscular inoculation with a virulent homologous rabies virus variant (EfV2) than wild caught bats. The primary inoculation of the less virulent homologous virus (EfV1) or a virulent heterologous variant (LnV1) did not appear more virulent in naïve bats [18]. However, the variability between this and previously published studies may be the use of separate RABV isolates, different inoculation techniques, and amount of virus in the inoculum [17], [18].

## Materials and Methods

### Animals

#### Ethics statement

Experimental design and animal care were done in compliance with the USDA animal care and welfare act (AWA) and the Association for Assessment and Accreditation of Laboratory Animal Care International (AAALAC). The use of bats in this experiment was approved and conducted in accordance with the Wadsworth Center IACUC.

#### Animals

Fifteen *E. fuscus* bats were born to adult females maintained in our captive colony. All adult bats had been tested for VNA and were found to be negative. Baby bats were raised to independence by their mothers. To identify individual bats, a colored band was placed on the forearm.

Bats were provided fresh water and ad lib mealworms daily. The room temperature was maintained at 85–90°F and approximately 60–80% relative humidity. Twice a week bats were given a brief physical exam, weighed, and an oral swab was obtained.

A bat that had lost 2 grams between weightings was placed in a smaller isolation cage, monitored more closely, hand-fed meal-worms and beef baby food, and if necessary, administered 0.5 ml lactated Ringers saline subcutaneously every 24 hours. If a bat was demonstrating clinical signs of rabies and did not improve within 24–48 hours it was euthanized, necropsied, tested for the presence of rabies virus antigen via the direct fluorescent antibody test (DFA); sera was collected to assay for rabies VNA [19].

### Serology

Seven days post RABV inoculation, then every two weeks, bats were bled from the uropatagial vein. Blood was collected in heparinized microcapillary tubes following veinpuncture with a 26 gauge needle. Samples remained at 4°C overnight and were separated via centrifugation the following day [20]. Using the tissue culture serum neutralization test protocol (TCSN), samples were processed as described in Trimarchi et al 1996 [19]. The virus employed in this assay was the Challenge Virus Strain (CVS-11). The assay was modified to reduce the amount of serum required. The final volume of rabies immune globulin (RIG) (Laboratory of Standards and Testing, Food and Drug Administration, Silver Spring, MD USA), positive and negative controls, test bat serum, was 25 ul instead of 50 ul. This micro-method procedure has a limit of detection sensitivity of 0.125 IU of rabies neutralizing antibody. Final antibody titer results were calculated taking into consideration the altered volumes of the titrations and the initial dilution of the test sample. Five RIG dilution replicates were performed on each TCSN and the bat sera results were compared to the RIG titer. Dilution end point titers were converted to IU/ml as based on the US standard RIG results for each test.

### Viruses

Virus was isolated from the salivary glands of two *E. fuscus* bats, designated as EfV1 and EfV2 [18], and a *L. noctivagans* bat (LnV1), as described in Rudd and Trimarchi (1987, 1989) [21]. To obtain adequate amounts of virus for inoculation, the isolates were passed three times in neuroblastoma (NA) cells (C-1300). To confirm the rabies virus variant, the N gene of virus isolate was sequenced as previously described [18]. For any bat that was positive for rabies virus infection via the DFA, the infecting RABV was confirmed utilizing PCR and sequence analysis.

### Inoculation Protocol

To prevent interference from maternal antibodies and concerns related to the immune system of juvenile animals, naïve bats were inoculated when they had reached 12 months of age. In a separate, previously published experiment, all wild caught bats inoculated with 10^4^ TCID_50_ EfV2 developed clinical rabies infection within 19 days post inoculation [18]. We anticipated a lower dose would allow us to follow the immune response and dissemination to the salivary glands. Bats were divided into three groups composed of 5 mixed gender individuals. Naïve bats were inoculated with 10^3^ TCID_50,_ in a volume of 50 ul in their right deltoid muscle with either EfV1, EfV2, or LnV1. Due to the lack of detectable VNA following the primary inoculation, the viral titer of the secondary challenge inoculation was increased. The second challenge inoculation occurred 238 days after the primary inoculation. Regardless of the primary inoculum, all surviving naive bats were administered a secondary challenge of 10^5^ TCID_50_ EfV2 in 50 ul in the right deltoid muscle. Table 1. To assess the significance between the groups, analysis was performed using a one way ANOVA.

**Table 1:** Bats were separated into three groups of five and inoculated i.m. with 10^3^TCID_50_ of either EfV1, EfV 2 or LnV1.

Group No.	No of bats	Virus/Titer	Challenge virus/Titer
Group 1	5	EfV1/10^3^TCID_50_	EfV2/10^5^TCID_50_
Group 2	5	EfV2/10^3^TCID_50_	EfV2/10^5^TCID_50_
Group 3	5	LnV1/10^3^TCID_50_	EfV2/10^5^TCID_50_

Bats were challenged with 10^5^TCID_50_EfV2 238 days after the first inoculation. Titer is based on the amount of virus in 50 ul of inoculum.

### Oral Swabs

Following RABV inoculation, oral swabs were collected twice week and immediately prior to euthanasia. Sterile urethral FLOQ Swabs ™ were inserted into the mouth of bats and applied to gum surfaces to absorb saliva. Swabs were then placed in 500 ul of Oral Swab Growth Media (OSGM) consisting of Eagles Minimum Essential Media supplemented with 10% fetal bovine serum, 2.0 mM glutamate, 100 IU penicillin G, 50 ug streptomycin, and 2.5 mg amphotericin B per ml. Oral swabs were tested for evidence of rabies virus via inoculation of NA cells and by real-time qPCR.

For viral isolation, 200 ul of the oral swab suspension was vortexed in a class II biological safety cabinet. Suspensions were centrifuged for 30 minutes at 4°C at 10,000 rpm. Supernatant (100 ul) was placed into a 1 ml microtube and 200 ul of NA cells at a concentration of 5×10^5^ per ml were added to the suspension. The tube was held at 4°C for 15–20 minutes and mixed by inversion every 5 min. One mL of OSGM was added to duplicate wells of a 24 well cell culture plate and one half of the inoculum/cell suspension was added to each well. Incubation was in a moist chamber at 34°C with 5% CO_2_ for 4 days.

After incubation, one well from each sample was passaged and seeded onto a new 24 well plate, in duplicate wells, and allowed to grow for four more days. The OSGM media was aspirated from the remaining well; cells were washed with PBS, pH 7.6 for 1 min., and fixed overnight with methanol-formalin fixative (1∶1 methanol and 10% formalin solution). Following fixation, two 30 minute PBS washes were applied to each well. Each well was washed independently to avoid cross contamination. Cells were stained with Light Diagnostics™ Rabies DFA Reagent (Cat.No. 5100, Chemicon International, Temecula, CA) for 30 minutes followed by two 2 minutes PBS washes. Prior to viewing on a Zeiss Axiovert 200 fluorescence microscope at 200 and 400X each well was flooded with 0.20 ml of 0.85% saline buffered with 0.05 m Trizma®, pH 9.0. The blind passaged plates were fixed, stained, and examined by fluorescent microscopy as described above.

RNA was extracted from the oral swab using 200 ul of sample added to Trizol LS reagent and processed per manufacturer’s recommendations (Invitrogen, Carlsbad, California). The cDNA was generated from extracted RNA as described in the Quanta qScript ™ cDNA Synthesis Kit (Quanta BioSciences, Gaithersburg, Maryland) using random primers. Quantification of viral RNA was accomplished via a TaqMan based quantitative reverse transcriptase polymerase chain reactions (qRT-PCR) as previously described in Nadin-Davis, 2009 [22] using the RABVD1 probe/primer set. A GFP mRNA assay was used as an internal extraction and positive control. The real time assay was performed using the qScrip Fast One-Step qRT-PCR Kit, Low ROX (Quanta, Gaithersburg, MD) per manufacturer’s recommendations. Cycling conditions were as follows: 50°C for 5 min, 95°C for 30 sec, and 45 cycles of 95°C for 15 sec and 50°C for 1 min. The assay was run on a fast ABI 7500 real time PCR system. Our LOD was 170 gene copies with a CT of 37. Analysis of results was performed using the ABI 7500 software.

## Results

### A Robust Antibody Response Following RABV Inoculation was not Detected in Naïve Bats

None of the naïve bats developed detectable VNA during the first 238 days of the study, including the terminal bleed. One bat (#14) inoculated with LnV1 developed VNA and developed clinical signs of rabies 20 days post inoculation (dpi) after the secondary inoculation with EfV2 (Table 2).

**Table 2:** Serological and survival results of naïve *E. fuscus* inoculated with a homologous or heterologous rabies virus variant.

Virus variant	Group	dose	Bat no	DFA	Incubation time in days pi	+ Oral swab in days pi	Infecting Variant	Rabies virus titer VNA (IU/ml) days post primary inoculation								
								7	21	49	*	245	259	287	315	364
EfV1	1	10^3^														
			1	**+**	**17** †	N	EfV2	<0.125^a^	<0.125	<0.125		<0.125	<0.125^b^			
			2	**+**	**16**	N	EfV2	<0.125	<0.125	<0.125		<0.125	<0.125^b^			
			3	+	64	N	EfV1	<0.125	<0.125	<0.125^b^						
			4	+	26	19,23	EfV1	<0.125	<0.125	<0.125^b^						
			5	**+**	**18**	N	EfV2	<0.125	<0.125	<0.125		<0.125	<0.125^b^			
																
EfV2	2	10^3^														
			6	+	16	16	EfV2	<0.125^b^								
			7	+	69	N	EfV2	<0.125	<0.125	<0.125^b^						
			8	+	19	N	EfV2	<0.125	<0.125^b^							
			9	+	22	N	EfV2	<0.125	<0.125^b^							
			10					<0.125	<0.125	<0.125		<0.125	<0.125	<0.125	<0.125	<0.125
																
LnV1	3	10^3^														
			11		**56**	N	EfV2	<0.125	<0.125	<0.125		<0.125	<0.125^b^			
			12		**26**	N	EfV2	<0.125	0.125^c^	<0.125		<0.125	<0.125^b^			
			13					<0.125	<0.125	<0.125		<0.125	<0.125	<0.125	<0.125	<0.125
			14		**20**	N	EfV2	<0.125	<0.125	<0.125		<0.125	0.5^b^			
			15	+	25	23	LnV1	<0.125	<0.125							

* Time point of 238 day challenge.

† Incubation times in bold are days post 238 challenge.

N = Negative.

a <0.125 indicates the results are below the limit of detection for our test.

b denotes terminal bleed.

c Demonstrated some neutralizing activity.

### *E. fuscus* was More Susceptible to a Homologous RABV

Four of the five naive bats (80%) inoculated with EfV2 developed rabies as compared to two of five naïve bats (40%) inoculated with EfV1, and one of five (20%) inoculated with LnV1. The differences in incubation times among these three groups of naïve bats were negligible. The mean incubation time for bats inoculated was 32 days post inoculation (dpi) (range 16–69 days) and 45 dpi (range 26–64 days) for EfV2 and EfV1, respectively. The incubation time for the one bat that developed rabies following inoculating with LnV1 was 25 dpi (Table 2).

### Prior Inoculation with RABV may not be Protective Following a Challenge Inoculation

The single naïve bat that survived the primary inoculation with EfV2 survived the secondary inoculation. However, all bats that survived the primary inoculation with EfV1 and three of the four surviving bats initially inoculated with LnV1 developed rabies following the secondary inoculation with EfV2. The mean incubation time for bats initially inoculated with EfV1 was 17 dpi (range 16–18 days) following the secondary inoculation with EfV2. The mean incubation time for bats previously inoculated with LnV1 was 31dpi (range 20–46 days) following the secondary inoculation with EfV2.

Of the seven naïve bats that developed rabies, five of the cases occurred within 26 dpi. The incubation period of two bats was considerably longer; 64 and 69 dpi. It is possible that these two bats had an additional exposure to RABV via a cage mate that was rabid. However, this may also be the result of inherent variation. Previous studies have reported incubation periods in excess of six months in bats [23], [24].

### Rabies Virus was Infrequently Recovered in the Oral Swabs of Naïve Bats

Few oral swabs collected from naïve bats following virus inoculation were positive for RABV RNA and/or live virus. Bat 6, inoculated with EfV2, began demonstrating clinical signs of rabies 16 dpi. An oral swab was collected immediately prior to euthanasia and was positive on virus isolation. Bat 4, inoculated with EfV1, demonstrated clinical signs of RABV infection 26 dpi. Viral RNA was extracted from an oral swab collected from bat 4 19 dpi and virus was isolated from a swab collected 23 dpi. Bat 15, inoculated with LnV1, developed clinical signs compatible with rabies virus infection 25 dpi. Rabies virus was isolated from a swab collected 23 dpi. All oral swabs collected following the secondary inoculation with EfV2 were negative for the presence of viral RNA and infectious virus (Table 2).

## Discussion

The study of RABV in natural hosts such as bats can be challenging due to lengthy quarantine requirements, age variation, husbandry concerns, genetic differences between animals, and unknown history of previous exposure to RABV. To date, all RABV studies in bats have been accomplished using wild caught animals. Determining the RABV exposure history of a wild caught bat based on the presence or absence of VNA may be misleading. Previous studies suggest the presence of VNA following natural or laboratory inoculation is often ephemeral [16], [17]. Furthermore, natural or experimental exposure to RABV does not always result in the production of VNA, even in the event of a fatal outcome [16], [17], [25]. Thus, the lack of VNA in wild caught bats may not be an accurate indicator of previous exposure to RABV.

The purpose of this study was to determine the susceptibility of naïve bats to homologous and heterologous RABV. Additionally, to better understand the virus-host interaction, one group of naïve bats was inoculated with a heterologous RABV. The heterologous RABV employed in this experiment, LnV1, is putatively more pathogenic in humans [9]–[12].

Our results suggest that VNA alone may not be the most critical determinant of rabies survival in bats. None of the naïve bats developed VNA at any point following the primary inoculation yet 53% of bats remained healthy following the first inoculation. Additionally, only one naïve bat developed detectable VNA (0.5IU) following challenge. Conversely, a study by Turmelle et al, 2010, reported that 92% of wild-caught, seronegative bats developed VNA following a primary i.m. RABV inoculation [17]. The discrepancy between these two studies may be the use of wild caught bats during the 2010 study. In bats, the development of protective, anti-rabies VNA may require multiple, non-lethal exposures to RABV throughout life, possibly commencing as juveniles. The absence of VNA in naïve bats following the primary inoculation may be due to the lack of previous exposure to RABV. Additionally, our results suggest that previous exposure to RABV cannot be determined by the presence or absence of VNA.

One caveat in assessing the presence of VNA is the potential effectiveness of the RABV employed in the neutralization assay. Previous studies assessing the VNA response have demonstrated optimal results may be achieved when the RABV to which the animal model is exposed is the same virus used in vaccine neutralization assays [26], [27]. Although most studies assessing the presence of VNA in bats following RABV inoculation use the CVS [16]–[19], the use of a heterologous virus may limit the accuracy of the test. Few bat studies employing a classical RABV challenge (genotype 1) have evaluated the presence of VNA utilizing a homologous RABV in the neutralization assay. [28], [29]. To evaluate the use of a homologous RABV in our neutralization assays, future studies will include a comparison between the use of a homologous RABV and the traditional challenge virus employed in neutralization tests, CVS-11.

Although neutralizing antibodies are important to survival following an exposure to RABV, survival may also depend on the variant to which the animal is exposed, the overall health of the animal, the innate immune response, and other immunological and genetic factors yet to be identified in bats. The importance of the infecting variant has been documented in the numerous studies comparing laboratory (attenuated) and street (wild type) RABV strains [1], [30]. This study demonstrates bats may be less likely to develop rabies following a secondary inoculation with a RABV to which it was previously exposed. Cross protection was not consistently achieved: 85% of bats that were initially inoculated with EfV1 or LnV1 and challenged with EfV2 developed rabies following the second inoculation. The lack of protection is likely to be multifactorial, including the use of a higher viral dose in the second inoculum, lack of previous exposure, and/or lack of cross protection to the challenging RABV. The modern RABV cell culture vaccines, which are derived from a single RABV variant, are cross protective against all known RABV (Genotype I) strains [27]. With this knowledge, one would anticipate that some level of cross protection would be afforded from the first inoculation. The increased amount of RABV in the second inoculation may have overwhelmed the immune response. Conversely, the VNA produced following the first inoculation may remain bound to RABV and thus unavailable in the levels required to prevent infection following the second inoculation. However, conclusions drawn from these results require temperance due to the small number of bats within each group.

The route of inoculation may play a role in outcome of disease. The principle route of RABV exposure in wild bats is unknown. Two previous studies reported that EBLV-1 and 2 could result in clinical infection following intramuscular or subcutaneous (s.c.) inoculation [28], [29]. Bats in our experiment were inoculated exclusively via the intramuscular route thus potentially affecting the outcome of this study.

In a 2011 review, Lafon [31] describes the interplay between the host, RABV, and the innate and humoral immune responses during RABV infections. The role of cytokines and chemokines in RABV infection may best be described as a conundrum. RABV may exploit these immune modulators to evade the immune system [32]–[34]. Conversely, cytokines and chemokines may influence the outcome of the infection by the duration of expression and activation of innate and humoral response [32]. Up regulation of genes associated with the innate immune response, including IFN a/b, and IL-6 occurred in mice following RABV inoculation [33]. The role of the innate immune system following RABV inoculation in bats is not well understood. The current lack of biologics available to study the innate immune system of bats precludes our ability to assess this response following RABV inoculation.

The fatality rate was higher in naïve bats following the secondary inoculation as compared to previous experiments [17], [18]. Because we altered the secondary inoculation dose in the naïve bat study, one cannot determine if the greater fatality rate was the result of an increased viral inoculum, lack of previous exposures, or both. Future studies will need to address these concerns.

The lack of infection in naïve bats inoculated with LnV1 was notable as the decreased pathogenicity of the LnV1 in naïve *Eptesicus fuscus* bats suggests that bats may be less likely to develop rabies following exposure to a heterologous RABV. As reported by Strickler et al, 2010 [35], heterologous RABV infections in *E. fuscus* is uncommon, putatively hindered by host species barriers. Furthermore, we demonstrated that exposure to a pathogenic, heterologous RABV does not necessarily confer the required immunity to prevent RABV infection following a subsequent exposure to a homologous RABV.

The lack of RABV in the oral swabs may be genuine or influenced by the twice a week collection. Because RABV is shed intermittently in the saliva, it is possible that daily collection could have improved our ability to detect RABV in the saliva. However, if the lack of RABV in the oral swabs is genuine, it would help explain the lack of large scale outbreaks of RABV in bat colonies. To maintain transmission cycle, RABV must be secreted in the saliva as lack of infectious RABV in saliva would result in a dead end host.

This is the first RABV study in bats in which the animals were born in captivity. The significance of this study is demonstrated by the lack of RABV infection among naïve bats inoculated with a homologous and heterologous RABV. Additionally, our results show the variation among RABV variants as it relates to pathogenicity in naïve *Eptesicus fuscus*. These results demonstrate that the pathogenicity of RABV in bats may be partially dependent on the RABV to which the animal is exposed.
